# Liquid Biopsy Biomarkers in Endometrial Cancer: Current Landscape and Future Perspectives

**DOI:** 10.3390/biom16060911

**Published:** 2026-06-19

**Authors:** Walter Giuseppe Giordano, Ludovica Pepe, Canio Martinelli, Valeria Zuccalà, Giuliana Ciappina, Massimiliano Berretta, Giuseppe Giuffrè, Vincenzo Fiorentino, Antonio Ieni

**Affiliations:** 1 PhD Program in Translational Molecular Medicine and Surgery, Department of Biomedical Sciences, Dental Sciences and Morpho-Functional Imaging, University of Messina, 98125 Messina, Italy; waltergiordano1997g@gmail.com (W.G.G.); ludopepe97@gmail.com (L.P.); vincenzo.fiorentino@unime.it (V.F.); 2 Anatomic Pathology Unit, Department of Human Pathology in Adult and Developmental Age “Gaetano Barresi”, University of Messina, 98125 Messina, Italy; valeria.zuccala@unime.it (V.Z.); giuseppe.giuffre@unime.it (G.G.); 3 Unit of Obstetrics and Gynecology, Department of Human Pathology of Adult and Childhood “Gaetano Barresi”, University of Messina, 98125 Messina, Italy; canio.martinelli@shro.org; 4 Sbarro Institute for Cancer Research and Molecular Medicine and Center of Biotechnology, College of Science and Technology, Temple University, 1900 N 12 St, Philadelphia, PA 19122, USA; 5 Section of Experimental Medicine, Department of Medical Sciences, University of Ferrara, 44121 Ferrara, Italy; giuliana.ciappina@unife.it; 6 Medical Oncology Unit, Department of Clinical and Experimental Medicine, University of Messina, 98125 Messina, Italy; mberretta@unime.it

**Keywords:** endometrial cancer, liquid biopsy, circulating tumor DNA (ctDNA), circulating tumor cells (CTCs), extracellular vesicles (EVs), microRNAs, artificial intelligence, clinical trials, precision oncology

## Abstract

Endometrial cancer is the most common gynecologic malignancy in developed countries and remains challenging in terms of risk stratification, treatment monitoring, and early detection of recurrence. Liquid biopsy provides a minimally invasive approach for the dynamic assessment of tumor-derived biomarkers and may complement tissue-based diagnosis and molecular classification. This narrative review summarizes current evidence on circulating biomarkers in endometrial cancer, including circulating tumor DNA (ctDNA), circulating tumor cells (CTCs), extracellular vesicles (EVs), circulating microRNAs, and tumor-educated platelets, with attention to validity, applicability, and implementation barriers. Among these biomarkers, ctDNA currently has the strongest evidence base, especially for longitudinal monitoring, prognostic stratification, molecular residual disease assessment, and early detection of relapse in high-risk or recurrent disease. However, its sensitivity remains limited in early-stage, low-volume, and low-shedding tumors. CTCs, EVs, microRNAs, and platelet-derived signatures are promising but still largely investigational. Artificial intelligence may support multimodal biomarker validation, although clinical adoption will require external validation, locked algorithms, standardized workflows, and prospective utility trials. Overall, liquid biopsy represents a promising adjunct to tissue-based diagnosis and molecular classification in endometrial cancer, particularly for monitoring and follow-up. Prospective studies are now needed to demonstrate whether liquid-biopsy-informed decisions can improve outcomes or safely reduce overtreatment.

## 1. Epidemiology and Burden of Disease

Endometrial cancer is the most prevalent gynecologic malignancy in developed countries and represents a growing global health burden. In 2022, approximately 420,000 new cases and nearly 98,000 deaths were estimated worldwide, with incidence increasing in many regions, particularly in high-income countries [1,2]. The rising burden is closely linked to population aging, declining fertility, obesity, diabetes, and other metabolic risk factors; obesity alone is estimated to account for a substantial fraction of cases in developed settings [2,3].

Most patients are postmenopausal, but 10–15% of cases occur in premenopausal women, and a smaller subset develops in the setting of hereditary predisposition, particularly Lynch syndrome [4,5]. Abnormal uterine bleeding often leads to early diagnosis, and most tumors are detected at an apparently uterus-confined stage. Nevertheless, outcomes remain poor in advanced, recurrent, high-grade, or biologically aggressive disease [3,5].

This epidemiologic and clinical heterogeneity creates a need for biomarkers that complement histopathology and tissue-based molecular classification. In this context, liquid biopsy is attractive not primarily as a replacement for diagnostic biopsy, but as a minimally invasive strategy for dynamic risk refinement, treatment monitoring, molecular residual disease assessment, and early detection of relapse [1,2,3,4,5].

### Literature Search Approach

A targeted narrative literature search was conducted to identify peer-reviewed studies and registered clinical trials on liquid biopsy biomarkers in endometrial cancer. PubMed/MEDLINE, Scopus, Web of Science, and Google Scholar were searched from database inception to 11 June 2026 using combinations of the following terms: endometrial cancer, endometrial carcinoma, uterine cancer, liquid biopsy, circulating tumor DNA, ctDNA, cell-free DNA, cfDNA, circulating tumor cells, extracellular vesicles, exosomes, circulating microRNA, tumor-educated platelets, molecular residual disease, recurrence monitoring, artificial intelligence, machine learning, and clinical trials. References of relevant articles were manually screened to identify additional primary studies. Eligible studies included original translational or clinical investigations, systematic reviews, meta-analyses, and high-quality reviews addressing analytical validity, prognostic value, longitudinal monitoring, tissue–plasma concordance, molecular classification, or clinical implementation of liquid biopsy in endometrial cancer. Studies focused exclusively on non-endometrial malignancies were excluded unless used as comparator evidence for clinically validated monitoring paradigms, such as ctHPV DNA in cervical cancer. ClinicalTrials.gov was searched using endometrial cancer/endometrial carcinoma combined with ctDNA, cell-free DNA, liquid biopsy, circulating tumor DNA, molecular residual disease, methylation, fragmentomics, cervical smear, urine, vaginal samples, recurrence, and monitoring. Because this article was designed as a narrative review rather than a systematic review, no formal risk-of-bias assessment, quantitative evidence grading, or PRISMA flow diagram was generated.

## 2. Pathogenesis and Molecular Landscape

Endometrial cancer arises through interactions among hormonal, metabolic, inflammatory, genetic, and environmental factors. The classical Bokhman model distinguishes estrogen-related type I tumors, usually low-grade endometrioid carcinomas, from type II tumors, including serous and clear cell carcinomas with more aggressive behavior [6]. Although clinically useful, this dualistic model does not capture the full molecular diversity of the disease.

The Cancer Genome Atlas redefined endometrial cancer into four molecular groups: POLE-ultramutated tumors, mismatch repair-deficient/microsatellite instability-high tumors, copy-number-low/no specific molecular profile tumors, and copy-number-high/p53-abnormal tumors [7]. These subgroups differ markedly in genomic architecture, immune contexture, prognosis, and therapeutic implications. POLE-mutated tumors usually have excellent outcomes despite high mutation burden, whereas p53-abnormal tumors are associated with aggressive behavior and poor prognosis [7,8].

Molecular classification has therefore become central to contemporary risk stratification and treatment planning. Current recommendations increasingly integrate molecular subtype with histotype, grade, myometrial invasion, lymphovascular space invasion, and stage to refine adjuvant therapy decisions [8,9].

Additional biological processes, including autophagy regulation and endometriosis-associated neoplastic transformation, also illustrate the biological heterogeneity of endometrial and endometriosis-related pathology [10,11].

However, tissue molecular profiling is usually performed at a single time point and may not fully capture temporal evolution, clonal selection, or treatment-emergent resistance. Liquid biopsy could complement tissue analysis by enabling serial assessment of tumor-derived material during follow-up and treatment [7,8,9].

## 3. Current Diagnostic and Prognostic Strategies

Diagnosis and management of endometrial cancer rely on clinical assessment, imaging, histopathology, surgery, and molecular profiling. Abnormal uterine bleeding, particularly after menopause, usually prompts evaluation. Transvaginal ultrasonography can be used as a triage tool, while histopathological confirmation is obtained by office endometrial biopsy, dilation and curettage, or hysteroscopy with directed biopsy when indicated [12,13,14].

After diagnosis, surgical staging and risk stratification guide treatment. Contemporary management integrates anatomic stage with histotype, grade, depth of myometrial invasion, lymphovascular space invasion, cervical involvement, and molecular subtype. The 2023 FIGO staging system and ESGO/ESTRO/ESP recommendations reflect this shift toward combined clinicopathological and molecular risk assessment [8,15,16].

Despite these advances, most diagnostic and prognostic information is still derived from tissue obtained at diagnosis or surgery. This remains indispensable, but it is not well suited to repeated, longitudinal assessment of tumor burden, molecular evolution, treatment response, or molecular residual disease [8,15,16].

## 4. Limitations of Current Diagnostic Approaches

Current tissue-based approaches provide essential diagnostic and prognostic information but have important limitations. A single biopsy or surgical specimen may underrepresent intratumoral heterogeneity, particularly in high-grade, mixed-histology, metastatic, or treatment-exposed tumors [17,18]. In addition, tissue analysis is static, whereas endometrial cancer can evolve through clonal selection and acquisition of resistance mechanisms during therapy [19].

Repeated tissue sampling is often impractical, invasive, costly, or technically impossible in recurrent or metastatic disease. Standard surveillance, based on symptoms, clinical examination, and imaging, may also detect relapse only after molecular recurrence has already occurred [19].

Liquid biopsy addresses some of these limitations by providing minimally invasive, repeatable access to tumor-derived molecular signals. Its interpretation must nevertheless be cautious, especially in early-stage disease where circulating tumor fractions can be very low [19].

## 5. Biological Basis and Technologies of Liquid Biopsy

Liquid biopsy refers to the analysis of tumor-derived material in biofluids, most commonly blood, but also urine, uterine aspirate/lavage, cervical/vaginal samples, and other fluids. Tumor-derived signals include ctDNA, CTCs, EVs, circulating RNAs, proteins, and platelet-associated signatures released through apoptosis, necrosis, active secretion, cellular turnover, and tumor-host interactions [19,20,21,22,23,24].

ctDNA is the most clinically mature analyte because it can carry tumor-specific mutations, copy-number changes, microsatellite instability-related signals, methylation patterns, and fragmentomic features. CTCs provide cellular and phenotypic information, while EVs, microRNAs, and tumor-educated platelets may capture additional layers of tumor biology and host response [22,23,24].

Analytical platforms include digital PCR for highly sensitive interrogation of known targets and next-generation sequencing for broader genomic profiling. Emerging approaches such as methylation analysis, fragmentomics, molecular barcoding, error suppression, microfluidics, and multimodal machine-learning models aim to increase sensitivity in low-burden settings [19,21,25].

**Figure 1:**
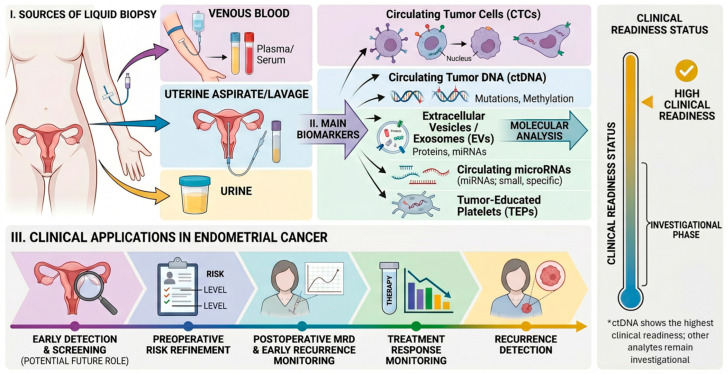
Overview of liquid biopsy in endometrial cancer. Liquid biopsy specimens can be obtained from venous blood, uterine aspirate/lavage, and urine. The main analytes include circulating tumor cells (CTCs), circulating tumor DNA (ctDNA), extracellular vesicles/exosomes, circulating microRNAs, and tumor-educated platelets. These biomarkers may support early detection and screening, preoperative risk refinement, postoperative minimal residual disease (MRD) and early recurrence monitoring, treatment response assessment, and recurrence detection. Among currently available analytes, ctDNA has the highest level of clinical readiness, whereas other biomarker classes remain predominantly investigational and require prospective validation and workflow standardization before routine clinical implementation. The initial illustrative image was generated using Google Gemini 3.1 Pro with the Nano Banana 2 image-generation model and was subsequently edited by the authors.

The key clinical question is not whether circulating tumor-derived material can be detected, but whether its detection improves patient management. This requires standardized pre-analytical workflows, validated assays, clinically meaningful thresholds, and prospective studies demonstrating utility [19]. A schematic overview of the main liquid-biopsy sources, circulating analytes, potential clinical applications, and relative clinical-readiness status in endometrial cancer is provided in Figure 1.

## 6. Circulating Biomarkers in Endometrial Cancer

The application of liquid biopsy in endometrial cancer has increasingly focused on the molecular characterization of circulating biomarkers, with the aim of capturing tumor heterogeneity and dynamic genomic evolution. Unlike tissue biopsy, circulating biomarkers reflect both spatial and temporal tumor diversity, making them particularly suitable for longitudinal disease monitoring. Among these, circulating tumor DNA (ctDNA), circulating tumor cells (CTCs), extracellular vesicles, microRNAs, and tumor-educated platelets represent the most extensively investigated components, each providing distinct but complementary molecular information [19,20,21,22,23,24].

### 6.1. Circulating Tumor DNA (ctDNA)

Circulating tumor DNA (ctDNA) is currently the most advanced liquid biopsy biomarker in endometrial cancer. It consists of tumor-derived fragments within the wider pool of cell-free DNA and may carry somatic mutations, copy-number alterations, microsatellite instability-related features, methylation changes, and fragmentation patterns. Plasma genomic studies in advanced endometrial cancer have detected recurrent alterations in TP53, PIK3CA, PTEN, ARID1A, KRAS, and clinically relevant copy-number events such as CCNE1 amplification, supporting ctDNA as a non-invasive surrogate of tumor genomics in higher-shedding disease [26].

A central issue is the rate of ctDNA positivity according to disease stage and biological risk. Because available studies use heterogeneous assays, sampling time points, tumor-informed versus tumor-naive designs, and positivity thresholds, a reliable pooled ctDNA positivity rate stratified by FIGO stage cannot yet be calculated. However, a consistent pattern is evident: ctDNA detection is substantially more frequent in high-grade, high-risk, bulky, advanced, or recurrent disease than in low-volume early-stage tumors. Ashley et al. detected baseline cfDNA mutations in 8 of 36 newly diagnosed patients (22%), with positivity confined to cases with advanced stage, high tumor volume, and/or aggressive histology [27]. Casas-Arozamena et al. detected ctDNA at surgery in 52 of 177 evaluable patients (29.4%), with enrichment in high-grade tumors, FIGO III-IV disease, deep myometrial invasion, lymphovascular invasion, and non-endometrioid or aggressive histologies [28]. Thus, negative ctDNA in early-stage disease should not be interpreted as biological absence of disease but rather as a limitation of blood-based detection in low-shedding settings.

Concordance between tissue and plasma is therefore conditional rather than absolute. Tumor-informed or tumor-guided assays generally perform best because they first define patient-specific tumor mutations and then interrogate plasma for those targets. In Ashley et al., when ctDNA was present and tumor mutations were covered by the assay, tissue–plasma concordance was high, with 35 of 38 baseline tumor mutations and 38 of 38 follow-up mutations detected in cfDNA [27]. Conversely, plasma-negative results in low-shedding disease should be interpreted as absence of detectable circulating tumor signal, not absence of tumor.

Longitudinal monitoring is the most compelling clinical application. Serial ctDNA levels can mirror treatment response, molecular residual disease, clonal evolution, and impending relapse. Moss et al. reported that matched and longitudinal plasma DNA analysis could detect recurrence or progression earlier than standard clinical assessment in selected patients and that ctDNA kinetics reflected treatment response [29]. Ashley et al. showed that ctDNA fractions and variant allele frequencies closely tracked response and progression, with lead time before clinical recurrence in selected cases [27]. Casas-Arozamena et al. reported recurrence detection before clinical confirmation in selected patients, with an average lead time of approximately 4.7 months, although single-target ddPCR missed some events [28]. Jamieson et al. found preoperative ctDNA mutations in 6 of 24 endometrial cancer patients (25%), associated with advanced stage and recurrence, and in selected patients ctDNA preceded clinical, radiological, or biomarker progression by 2–5 months [30].

These findings support ctDNA primarily for postoperative molecular residual disease assessment, recurrence surveillance, treatment-response monitoring, and risk refinement rather than stand-alone screening. This distinction is crucial because most endometrial cancers are diagnosed at an early stage after abnormal uterine bleeding, when blood-based ctDNA sensitivity may be limited. ctDNA is therefore more likely to add information by identifying biologically aggressive disease, occult residual disease, or impending relapse in already diagnosed patients than by replacing endometrial sampling, hysteroscopy, imaging, and histopathological assessment [13,14,15,27,28,29,30,31,32].

The cervical cancer experience with circulating HPV DNA illustrates both the promise and the challenge. In HPV-associated cervical cancer, ctHPV DNA benefits from a tumor-specific viral target usually absent from normal human DNA, and clinical validation after chemoradiation has shown strong prognostic value for residual disease detection [32]. Endometrial cancer lacks a universal viral marker and is molecularly heterogeneous; a comparable level of validation will likely require tumor-informed or molecular-subgroup-informed assays, standardized time points, predefined thresholds, correction for clonal hematopoiesis, and prospective trials showing that ctDNA-guided decisions improve outcomes rather than simply predict relapse.

Overall, ctDNA is the liquid biopsy analyte closest to clinical implementation in endometrial cancer. Its near-term rationale is strongest in high-risk, p53-abnormal, advanced, recurrent, or postoperative MRD settings. Key barriers remain low sensitivity in early-stage/low-shedding disease, pre-analytical variability, assay heterogeneity, and lack of interventional evidence. A focused synthesis of representative ctDNA studies is provided in Table 1 [27,28,29,30,31,32,33,34,35].

### 6.2. Circulating Tumor Cells (CTCs)

Circulating tumor cells (CTCs) represent a complementary component of liquid biopsy because, unlike acellular analytes, they enable direct phenotypic and molecular interrogation of intact tumor cells released from primary and/or metastatic sites. In endometrial cancer, the available evidence indicates that CTCs comprise biologically heterogeneous populations with epithelial, mesenchymal, and intermediate plastic states, consistent with the dynamic nature of metastatic dissemination [36,37,38]. Molecular profiling of EC-derived CTCs has demonstrated enrichment for EMT- and stemness-associated programs, including ETV5, NOTCH1, SNAI1, TGFB1, ZEB1/2, ALDH, and CD44, together with expression changes involving pathways relevant to EC progression, such as PI3K-related and Wnt/β-catenin signaling.

Clinically, although the available studies remain limited by relatively small cohorts, CTC detection has been associated with adverse pathological features, including deep myometrial invasion, lymph node involvement, and cervical extension, and preliminary serial data suggest that CTC dynamics may also reflect treatment-related changes. At the same time, the low abundance of CTCs in peripheral blood, their marked phenotypic heterogeneity, and the lack of standardized enrichment and detection platforms continue to limit analytical sensitivity and broader clinical implementation, particularly in early-stage disease. Notably, the detection of CTCs in ovarian vein samples from patients with apparently localized disease further suggests that conventional peripheral blood assays may underestimate tumor dissemination. Overall, CTCs are a biologically informative analyte in endometrial cancer and hold promise for risk stratification, disease monitoring, and the study of metastatic progression; however, their routine clinical use will require larger prospective validation studies and more robust, standardized detection strategies [39,40,41].

### 6.3. Extracellular Vesicles and Exosomes

Extracellular vesicles (EVs), particularly exosomes, represent a functionally active component of the liquid biopsy landscape. Released by tumor, stromal, endothelial, and immune cells, EVs mediate intercellular communication and carry DNA, mRNA, microRNAs, long non-coding RNAs, proteins, and lipids that may reflect tumor biology and tumor–microenvironment interactions [23,42,43,44].

In endometrial cancer, preclinical and early translational studies have identified candidate EV-associated RNA and protein signatures with potential diagnostic and prognostic relevance [44,45]. Their lipid bilayer protects molecular cargo from degradation, making EVs attractive for biomarker discovery and potentially complementary to ctDNA, CTCs, and circulating miRNAs. However, current evidence remains exploratory, and immediate clinical translation is limited by EV heterogeneity, non-standardized isolation and characterization methods, and difficulty in confidently distinguishing tumor-derived vesicles from vesicles released by non-neoplastic cells. Accordingly, EVs should currently be regarded as a promising but investigational component of multimodal liquid biopsy strategies in endometrial cancer [23,42,43,44].

### 6.4. Circulating MiicroRNAs

Circulating microRNAs (miRNAs) are small non-coding RNAs released in extracellular vesicles, protein complexes, or from dying cells. Their stability in plasma and serum and their role in pathways such as PI3K/AKT/mTOR, Wnt/beta-catenin, TGF-beta signaling, and p53-mediated cell-cycle control make them attractive biomarker candidates in endometrial cancer [23,45,46].

Several miRNAs, including members of the miR-200 family, miR-21, miR-155, miR-182, miR-183, miR-205, and let-7 family members, have been linked to tumor progression, epithelial–mesenchymal transition, immune signaling, PTEN/PI3K pathway regulation, and aggressive clinicopathological features [46,47,48,49,50,51,52,53]. Diagnostic performance is generally stronger for multi-miRNA panels than for individual markers, with a meta-analysis reporting pooled sensitivity of 0.84, specificity of 0.87, and an area under the curve of 0.91 [50].

Despite these promising findings, circulating miRNA studies remain heterogeneous with respect to specimen type, normalization strategy, detection platform, and clinical endpoints. Non-tumor sources, especially blood cells and platelets, are important confounders. At present, miRNAs should be considered investigational and most likely to add value as part of multimodal panels rather than as stand-alone clinical tests [45,46,54,55].

### 6.5. Tumor-Educated Platelets

Tumor-educated platelets (TEPs) represent an emerging liquid biopsy component that reflects the interaction between tumor cells and the host systemic environment. Beyond their role in hemostasis, platelets can uptake tumor-derived extracellular vesicles, RNA, proteins, and cytokine-induced signals, resulting in altered RNA content, splicing patterns, protein composition, and functional behavior [24,56,57,58].

From a clinical perspective, TEP-based assays have shown promising diagnostic performance across several tumor types. In endometrial cancer, however, the evidence is still limited to exploratory studies. A diagnostic study combining platelet RNA sequencing with artificial intelligence showed promising discrimination between healthy individuals and patients with endometrial cancer, while separation between malignant and benign gynecologic conditions was more modest [59]. TEPs are abundant and may integrate signals from both tumor cells and the host microenvironment, but their clinical implementation is constrained by the lack of standardized protocols for platelet isolation, transcriptomic analysis, and control of confounding factors such as inflammation, comorbidities, and non-neoplastic systemic conditions. Therefore, TEPs should currently be considered an investigational platform rather than a clinically established biomarker in endometrial cancer [24,56,57,58,59].

### 6.6. Clinical Positioning of Liquid Biopsy Analytes in Endometrial Cancer

While the previous sections have examined individual liquid biopsy analytes from a biological and technical perspective, their clinical relevance ultimately depends on their ability to inform specific decision-making scenarios [19,20,21,22,23,24,25].

In this context, we propose a pragmatic, hypothesis-generating framework that positions the principal liquid biopsy analytes according to the clinical settings in which they may provide the most informative contribution. Rather than representing a prescriptive or guideline-based classification, this approach is intended to support interpretation of the current evidence by integrating considerations of clinical applicability, strength of available data, and level of translational maturity [26,27,28,29,30,31,32,33,34,35,56,57,58,59].

Among the available biomarkers, circulating tumor DNA (ctDNA) currently shows the most consistent and robust evidence, particularly in the settings of postoperative risk assessment and recurrence monitoring. In contrast, other analytes, including circulating tumor cells (CTCs), microRNAs, and extracellular vesicles, remain largely investigational, with heterogeneous and still limited clinical validation. Nevertheless, these biomarkers may provide complementary information and could become particularly relevant within integrated, multimodal strategies [27,28,29,30,31,32,33,34,35,38,39,40,41,42,43,44,45,46,47,48,49,50,51,52,53,54,55,56,57,58,59].

Importantly, the proposed positioning should be interpreted with caution, as the field is rapidly evolving and high-quality prospective validation studies are still needed before widespread clinical implementation can be considered. The proposed framework is summarized in Table 2 [26,27,28,29,30,31,32,33,34,35,56,57,58,59].

To complement this clinical positioning and directly address the current evidence base for ctDNA, Table 1 provides a ctDNA-focused synthesis of representative studies evaluating detectability, tissue–plasma concordance, molecular residual disease, and longitudinal monitoring in endometrial cancer.

### 6.7. Molecular Subgroup and Histotype-Specific Interpretation of Liquid Biopsy in Endometrial Cancer

Liquid biopsy results in endometrial cancer should be interpreted in a molecular-subgroup and histotype-specific framework. POLE-mutated, MMR-deficient, p53-abnormal, and NSMP tumors differ in prognosis, mutation burden, immune microenvironment, genomic instability, and shedding probability [8,60]. The same plasma result may therefore have different biological and clinical meaning across subtypes.

p53-abnormal and serous-like tumors are the most plausible setting for informative plasma ctDNA because they often show aggressive biology, high tumor burden, chromosomal instability, and recurrence risk. In these tumors, ctDNA may support longitudinal monitoring, early relapse detection, and identification of resistance mechanisms [26]. By contrast, low-grade endometrioid and many NSMP tumors are frequently uterus-confined and low-shedding; a negative plasma result should not alter diagnostic or surveillance decisions outside validated protocols.

MMR-deficient tumors offer opportunities for monitoring during immunotherapy, where ctDNA kinetics may reflect response or resistance, but endometrial-cancer-specific evidence remains limited. POLE-mutated tumors pose a different challenge: even if tumor-derived alterations are detectable, their excellent prognosis means that analytical positivity does not automatically imply therapeutic escalation [60].

These considerations reinforce that ctDNA complements rather than replaces tissue molecular classification. Tumor-informed assays, alternative biofluids such as uterine aspirate or cervical/vaginal samples, and multi-analyte strategies may be particularly valuable in low-shedding disease [27,28,61].

## 7. Clinical Integration and Future Perspectives

Liquid biopsy is most likely to enter endometrial cancer care as a complementary monitoring platform rather than as a replacement for histopathology or tissue-based molecular classification. The most realistic near-term uses are postoperative MRD assessment, recurrence surveillance, treatment-response monitoring, and molecular profiling when repeat tissue biopsy is not feasible [19,26,27,28,29,30,31,32,33,34,35].

Clinical application should be calibrated to disease setting. In advanced, recurrent, high-grade, or p53-abnormal disease, ctDNA is more likely to be informative. In early-stage, low-grade, or low-volume disease, blood-based sensitivity is limited; negative results should not change management unless validated in prospective protocols. Future utility will probably depend on integrating tissue molecular classification, ctDNA kinetics, other circulating analytes, imaging, histopathology, and clinical variables.

### 7.1. Artificial Intelligence, Computational Validation, and Clinical Adoption

Artificial intelligence (AI) and machine-learning methods may accelerate the validation and clinical adoption of liquid biopsy biomarkers by converting high-dimensional molecular signals into interpretable risk estimates. In endometrial cancer, the most relevant inputs include ctDNA variant allele fractions and kinetics, methylation or fragmentomic features, miRNA and EV signatures, platelet RNA profiles, tissue molecular subtype, histopathological risk factors, imaging, CA-125 where applicable, treatment exposure, and longitudinal outcomes [59,61,62].

The practical value of AI is not simply improved classification accuracy. Properly designed models could support sample quality control, detection of weak multimodal signals in low-shedding disease, recognition of clonal hematopoiesis or pre-analytical artifacts, individualized surveillance schedules, risk enrichment for clinical trials, and standardized multidisciplinary reporting. Longitudinal models may be especially useful for distinguishing true molecular relapse from stochastic low-level assay noise [59,61,62,63,64,65].

However, AI should be treated as a validation and implementation tool rather than a shortcut to clinical use. Models trained on small retrospective cohorts are vulnerable to overfitting, batch effects, spectrum bias, class imbalance, and poor transportability across laboratories. Before clinical adoption, algorithms should be locked before validation, tested externally in multicenter cohorts, calibrated to clinically meaningful thresholds, assessed with decision-curve and health-outcome analyses, and reported according to CONSORT-AI and SPIRIT-AI guidance [63,64].

### 7.2. Registered Clinical Trials and Prospective Translational Studies

Clinical adoption will require prospective studies that move beyond analytical validity to clinical utility. A ClinicalTrials.gov search identified completed, recruiting, and planned studies that explicitly evaluate ctDNA, cfDNA, DNA methylation, or other liquid-biopsy strategies in endometrial cancer. The most relevant studies address tissue–plasma concordance, postoperative MRD, recurrence-risk stratification, ctDNA-guided adjuvant selection, treatment-duration decisions in advanced/recurrent disease, and early detection using non-blood biofluids or cfDNA features (Table 3) [66].

Importantly, registry-based evidence should be interpreted cautiously. Enrollment, status, endpoints, and assay details may change over time, and most trials are designed to establish feasibility, prognostic value, or risk stratification rather than immediate practice-changing utility. The decisive next step will be showing that liquid-biopsy-informed interventions improve outcomes, safely reduce overtreatment, or optimize surveillance without increasing unnecessary imaging, anxiety, or cost [66].

### 7.3. Liquid Biopsy in Fertility-Sparing Management, EIN/AEH, and Early Endometrioid Disease

Fertility-sparing management and early endometrial neoplasia represent attractive but highly challenging settings for liquid biopsy. Patients with EIN/AEH or grade 1, stage IA endometrioid carcinoma are monitored with repeated endometrial sampling during conservative progestin-based treatment, and a non-invasive biomarker for early non-response or recurrence would be clinically useful [67,68,69,70,71].

At present, however, circulating biomarker sensitivity is expected to be low because these lesions are often small, low-grade, and low-shedding. ctDNA may therefore be uninformative in many patients, while alternative biofluids such as uterine aspirate, cervical/vaginal samples, and urine, or multimodal panels including methylation and miRNA features, may be more plausible research directions [61,69].

No liquid biopsy assay currently replaces histopathology for diagnosis, response assessment, or surveillance in EIN/AEH or fertility-sparing management. Future studies should use tumor-informed longitudinal designs, predefined time points, and clinically meaningful endpoints such as early detection of non-response, progression, or relapse [67,68,69,70,71].

### 7.4. Integrating Liquid Biopsy into the Pathology Workflow

The clinical value of liquid biopsy will depend on integration into a pathology-centered workflow. Histopathology remains the foundation for diagnosis, grading, histotype assignment, staging context, and molecular classification; liquid biopsy should be interpreted as an additional dynamic layer rather than an alternative to tissue evaluation [72,73].

A practical workflow would start with tissue diagnosis and molecular classification, followed by liquid biopsy only when it addresses a defined clinical question: postoperative MRD, suspected recurrence, longitudinal monitoring in advanced disease, or molecular profiling when new tissue is unavailable. Interpretation should incorporate pre-test probability, tumor subtype, burden of disease, treatment context, and assay limitations [72,73,74,75].

Discordant tissue–plasma findings require careful review. Tissue-positive/plasma-negative results are common in low-shedding disease and should not exclude tumor. Plasma-only alterations may reflect spatial heterogeneity, clonal evolution, or non-tumor sources such as clonal hematopoiesis; matched white-blood-cell sequencing may be needed in selected cases [20,74].

Implementation also requires harmonized pre-analytical protocols, validated analytical performance, traceable reporting, and multidisciplinary interpretation. In practice, liquid-biopsy findings in endometrial cancer should be reported together with histology, immunohistochemistry, tissue molecular classification and, when available, cytology-based or other ancillary molecular data, since experience with cytological molecular testing, immunohistochemical biomarker assessment and molecular surveillance in other tumor settings indicates that clinically useful information is strongest when interpreted within an integrated pathology workflow rather than as an isolated molecular readout [75,76,77,78,79].

## 8. Conclusions

Liquid biopsy is emerging as a clinically relevant adjunct in endometrial cancer, especially for dynamic disease assessment after diagnosis. Among available analytes, ctDNA has the most mature evidence base for longitudinal monitoring, postoperative molecular residual disease assessment, prognostic stratification, and early relapse detection in high-risk or recurrent disease. Its limited sensitivity in early-stage, low-volume, and low-shedding tumors means that ctDNA should not replace histopathological diagnosis or tissue-based molecular classification.

The most rational implementation path is subgroup-aware and tumor-informed. Tissue molecular classification should define the biological context and patient-specific targets, while serial liquid biopsy testing should be used where circulating shedding is likely to be informative. CTCs, EVs, microRNAs, and TEPs may add complementary biological information, but they remain investigational and require standardized workflows and external validation.

AI and multimodal modeling may help transform complex liquid biopsy data into clinically usable tools, but only if algorithms are transparent, externally validated, prospectively evaluated, and linked to actionable decisions. The growing number of registered clinical studies indicates that the field is moving toward ctDNA-guided monitoring and treatment strategies. If prospective trials demonstrate improved outcomes or safe reduction of overtreatment, liquid biopsy could become an important component of precision oncology in endometrial cancer.

## Figures and Tables

**Table 1:** ctDNA-focused synthesis of representative studies addressing detectability, tissue–plasma concordance, molecular residual disease, and longitudinal monitoring in endometrial cancer.

Study/Year	Cohort/Setting	Assay/Sample	Detectability/Stage Signal	Tissue–Plasma Concordance	Longitudinal/MRD Evidence	Clinical Interpretation
**Moss et al. [29], 2020**	Endometrial cancer patients with matched and longitudinal plasma samples	Targeted NGS/plasma cfDNA	Detection was most informative in patients with measurable or recurrent/progressive disease.	Matched plasma analysis captured tumor-related alterations and MSI changes in selected cases.	ctDNA kinetics reflected treatment response and enabled earlier detection of recurrence/progression in selected patients.	Supports feasibility of monitoring, but requires validation in larger prospective cohorts.
**Ashley et al. [27], 2023**	Prospective newly diagnosed EC cohort with serial sampling	Tumor NGS and high-depth cfDNA NGS with molecular barcoding	Baseline cfDNA mutations in 8/36 patients (22%); positivity concentrated in advanced, bulky, or aggressive disease.	When ctDNA was present and tumor mutations were covered, concordance was high: 35/38 baseline and 38/38 follow-up mutations detected.	ctDNA fraction/VAF mirrored response or progression and preceded clinical recurrence in selected cases.	Directly addresses feasibility, concordance, and longitudinal monitoring; sensitivity remains limited in low-burden disease.
**Casas-Arozamena et al. [28], 2024**	Localized and recurrent EC with plasma, uterine aspirate, surgical samples, and follow-up	Tumor/uterine-aspirate-informed sequencing and ctDNA tracking	ctDNA detected at surgery in 52/177 patients (29.4%); enriched in high grade, FIGO III-IV, deep invasion, and LVSI.	Tumor-informed design improved interpretability, but detectability depended on shedding and assay target selection.	Longitudinal ctDNA anticipated relapse in selected patients with an average lead time of ~4.7 months; single-target ddPCR missed some events.	Strong translational evidence for risk stratification and follow-up; assay harmonization is still needed.
**Recio et al. [31], 2024**	Post-surgical stage I uterine malignancies, including but not restricted to EC	Tumor-informed ctDNA MRD testing/blood	Designed for apparently localized disease after surgery, where standard risk factors may underrepresent residual risk.	Tumor-informed approach supports patient-specific tracking.	Postoperative ctDNA positivity identified patients at increased recurrence risk.	Promising MRD application, but uterine-malignancy cohort composition limits direct EC-only inference.
**Lindemann et al. [33], 2025**	International multicenter EC cohort	Preoperative plasma ctDNA	Preoperative ctDNA positivity associated with aggressive clinicopathological features and recurrence risk.	Supports plasma detection of clinically relevant tumor-derived signal in higher-risk cases.	Prognostic association with recurrence and survival outcomes.	Supports risk refinement; clinically actionable thresholds require prospective validation.
**Jamieson et al. [30], 2025**	Selective ctDNA testing in endometrial and ovarian carcinomas	Targeted ctDNA testing/plasma	Preoperative ctDNA mutations in 6/24 EC patients (25%), associated with advanced stage and recurrence.	Testing was most useful when selected by clinical and molecular context.	ctDNA preceded clinical, radiological, or biomarker progression by 2–5 months in selected patients.	Supports selective use for disease monitoring; broader validation is required.
**Ahmed et al. [34], 2026**	Systematic review and meta-analysis of perioperative ctDNA in EC	Evidence synthesis of pre- and postoperative ctDNA	Perioperative ctDNA positivity varied across studies, reflecting differences in assay and patient mix.	Not applicable; study-level synthesis highlights assay heterogeneity.	Both preoperative and postoperative ctDNA positivity were associated with worse progression-free survival; postoperative positivity showed strong prognostic relevance.	Confirms prognostic signal but underscores need for prospective interventional trials.

Abbreviations: cfDNA, circulating cell-free DNA; ctDNA, circulating tumor DNA; EC, endometrial cancer; MRD, molecular residual disease; MSI, microsatellite instability; NGS, next-generation sequencing; VAF, variant allele fraction.

**Table 2:** Positioning of liquid biopsy analytes across clinically relevant scenarios in endometrial cancer.

Clinical Scenario	ctDNA	CTCs	Extracellular Vesicles	Circulating microRNAs	Tumor-Educated Platelets	Practical Take-Home Message
Preoperative risk refinement	Most promising analyte for identifying biologically aggressive disease and plasma-detectable molecular alterations	Potentially informative, but currently limited by low abundance and lack of standardization	Exploratory	Exploratory; may support panel-based discrimination	Exploratory	ctDNA is currently the most clinically plausible blood-based adjunct for preoperative risk refinement
Postoperative MRD/early recurrence monitoring	Strongest current evidence; most realistic near-term application	Limited evidence	Not established	Investigational	Investigational	MRD and recurrence monitoring are the clearest near-term clinical roles for liquid biopsy in EC, mainly through ctDNA
Advanced/recurrent disease longitudinal monitoring	Useful for clonal evolution, treatment response, and resistance tracking	May provide complementary information on dissemination biology	May add biologic information on tumor–microenvironment signaling	Potential complementary role in dynamic monitoring	Limited evidence	Multimodal approaches may be most informative in advanced disease, but ctDNA remains the anchor analyte
Early-stage/low-shedding disease	Sensitivity may be limited; negative results must be interpreted cautiously	Limited sensitivity	Potential complementary role	Potential complementary role, especially in panel-based approaches	Uncertain	A negative blood test does not exclude disease in low-burden EC
Biologic characterization beyond tissue snapshot	Captures tumor-derived genomic evolution	Captures cellular plasticity and dissemination phenotype	Captures intercellular communication and tumor–microenvironment interactions	Captures regulatory and host–tumor interaction signals	Captures systemic host-response signatures	Different analytes provide different biological layers; they should be viewed as complementary rather than interchangeable
Current translational maturity	Highest	Low	Low	Low-to-moderate, but still investigational	Very low	ctDNA is closest to clinical implementation; the others remain primarily investigational

**Table 3:** Selected ClinicalTrials.gov-registered studies explicitly evaluating liquid biopsy, ctDNA/cfDNA, DNA methylation, or molecular monitoring strategies relevant to endometrial cancer.

NCT ID/Recruitment Status/Study Type	Population/Setting	Liquid Biopsy Analyte or Sample	Design/Primary Aim	Clinical Utility Addressed	Interpretive Caution
**NCT04456972/Completed/Interventional**	Patients with endometrial cancer during treatment	ctDNA compared with tumor tissue molecular analysis	Pilot study evaluating concordance between tumor-tissue molecular analysis and circulating tumor DNA	Tissue–plasma concordance and feasibility of ctDNA monitoring	Pilot sample size; not designed to establish ctDNA-guided management.
**NCT05049538/Recruiting/Observational**	High-risk endometrial cancer	Blood-based liquid biopsy with tumor molecular profiling	Determine utility of liquid biopsies and tumor profiling for predicting recurrence	Recurrence prediction and integration with tumor profiling	Single-institution translational design; clinical-action thresholds remain to be defined.
**NCT05955079/Recruiting/Observational**	Stage I-III endometrial cancer undergoing surgery	Pre- and postoperative ctDNA	Prospective biological collection assessing association between ctDNA detection and metastatic relapse risk	Postoperative MRD, recurrence-risk stratification, and surveillance utility	Prognostic study; interventional utility will require subsequent trials.
**NCT05504161/Unknown status/Observational**	Patients with endometrial cancer	Cervical swab tumor DNA and whole-blood ctDNA	Evaluate tumor DNA detection and prognostic/predictive value using cervical and blood-based sampling	Non-blood and blood-based detection and molecular characterization	Performance may differ by stage, histotype, and tumor shedding.
**NCT06341855/Recruiting/Interventional**	High-risk endometrial carcinoma after treatment	ctDNA-MRD	Explore prognostic and recurrence-monitoring value of ctDNA-MRD	MRD-based surveillance and early recurrence detection	Exploratory scale; assay and time-point standardization remain central.
**NCT06083779/Recruiting/Observational**	High-risk populations for endometrial cancer	Plasma cfDNA fragmentomics and multimodal cfDNA features	Develop non-invasive early detection of endometrial cancer	Addresses early-stage/low-burden detection challenge	Screening/early detection requires external validation and specificity in symptomatic populations.
**NCT07339384/Not yet recruiting/Interventional**	Stage I HIR endometrial cancer after surgery	Tumor-informed Signatera ctDNA assay	Assess whether ctDNA can guide adjuvant selection; ctDNA-negative observation compared with vaginal brachytherapy	ctDNA-guided adjuvant de-escalation/selection	Practice-changing only if recurrence outcomes and safety of de-escalation are demonstrated.
**NCT07270666/Recruiting/Interventional**	Advanced or recurrent MMRd/MSI-H endometrial cancer after chemotherapy plus ICI	Serial ctDNA	Evaluate ctDNA testing to inform continuation of standard-of-care treatment	ctDNA-guided treatment-duration decisions in immunotherapy-sensitive disease	Pilot design and selected molecular subgroup.
**NCT06846775/Recruiting/Observational**	Patients with diagnosed EC and healthy controls	DNA methylation in patient-collected urine and vaginal samples	Case–control evaluation of diagnostic utility of methylation testing	Patient-friendly detection using non-blood liquid biopsy samples	Case–control performance may overestimate real-world diagnostic accuracy.
**NCT07400835/Enrolling by invitation/Observational**	Women with postmenopausal bleeding	DNA methylation in urine, vaginal, and cervical samples	Compare methylation testing with traditional TVUS-based diagnostic pathway and assess 2-year EC risk	Risk stratification in symptomatic women and early detection pathway design	Requires careful comparison with histopathology and guideline-based workup.

Abbreviations: cfDNA, circulating cell-free DNA; ctDNA, circulating tumor DNA; EC, endometrial cancer; HIR, high-intermediate risk; ICI, immune checkpoint inhibitor; MMRd, mismatch repair deficient; MRD, molecular residual disease; MSI-H, microsatellite instability-high; TVUS, transvaginal ultrasonography.

## Data Availability

No new data were created in this study.
